# Variations in Hospital Admissions of Non-Communicable Disease Patients Before and During The COVID-19 Pandemic (A Tertiary Care Setting, January 2018–June 2021)

**DOI:** 10.1007/s44197-023-00174-5

**Published:** 2024-01-08

**Authors:** Seyma Aliye Kara, Banu Cakir

**Affiliations:** 1 Pursaklar District Health Directorate, Republic of Turkey Ministry of Health, Ankara, Turkey; 2 Division of Epidemiology, Department of Public Health, Hacettepe University Faculty of Medicine, Ankara, Turkey

**Keywords:** COVID-19, Noncommunicable diseases, Hospital admission

## Abstract

**Background:**

This study aimed to analyze the variations (if any) in hospital admissions of patients with any of the five common non-communicable diseases (NCDs), based on secondary analysis of electronic health records of patients admitted to Hacettepe University Hospitals at least once, from January 1, 2018 through June 15, 2021.

**Design:**

Data were recruited from hospital’s electronic health records on patients with diagnoses of ischemic heart disease, hypertension, diabetes, cancer, and chronic obstructive pulmonary disease, using relevant ICD-10 codes.

**Results:**

Compared to the corresponding time span in the pre-pandemic period, the number of hospital admissions of patients with selected five NCDs significantly decreased during the pandemic, with an official start in Turkey on March 11, 2020. Number of total-, out-patient-, and in-patient admissions of NCD patients were significantly lower in the pandemic period compared to the expected values in time series analysis, controlling for patient characteristics, and seasonality.

**Conclusions:**

Study findings suggest that there has been a prominent impediment in NCD patients’ access to, and/or use of health care services over the pandemic, which might evolve to higher admission rates, severity and fatality of such patients in the upcoming years. Further studies are warranted for confirmation of our findings in other care settings, with individual-based data on care compensation through settings other than regular admission sites (if any), and/or the reasons for under-use of services.

## Background

COVID-19 pandemic has been a major public health concern due to its devastating acute health effects in populations, with its potential for long-term complications. The “Long COVID-19” has been identified as a potential damage caused by SARS-CoV-2 presenting in up to one-third of infected individuals with persistent shortness of breath, chest pain, fatigue, headache, brain fog and palpitations [1]. Long COVID-19 has been linked with remarkable reduction in workforce due to disturbed health condition of those infected [2]. Worsening of several systemic diseases after SARS-CoV-2 infection have also increased the morbidity toll in public, during the pandemic, whilst new diagnoses of pulmonary fibrosis, myocarditis, diabetes, and stroke have been linked with COVID-19 infections [3]. With the accumulating evidence, there is a serious global concern that COVID-19 may lead to long-term adverse impact on public health, on cardiovascular health and mortality, in particular [1]. It is noteworthy that the unprecedent impact of COVID-19 on health service delivery might have led to individual or institutional delays (or neglect) in proper management of noncommunicable diseases over the pandemic [4]. Inability to receive timely, appropriate, and adequate care by patients with NCDs (if any) will affect the prognosis of such diseases over the years, which may trigger a decrease in disability-adjusted life expectancy in societies in upcoming years.

Close monitoring of patients with non-communicable diseases (NCD) for direct adverse effects of COVID-19 infection, and for potential complications/disability in these patients due to improper care over the pandemic is essential for appropriate adjustments in future action plans for NCDs [5].

This study aimed to investigate the variation in NCD patients’ admissions to a tertiary care hospital over the pandemic, as a surrogate measure for the impact of COVID-19 on NCD resources and services. A retrospective analysis of hospital’s electronic health records were conducted to investigate all NCD patient admissions over 3.5, starting from January 1, 2018. Data on patients with any of the five major NCDs (namely, ischemic heart disease, hypertension, diabetes, cancer, and chronic obstructive lung disease) were recruited for detailed analyses of admission type (in- or outpatient), frequency, distribution by clinical departments, and time. Admissions were compared and contrasted taking March 11, 2020 as the official start of the pandemic in the country and all analyses were controlled for age, gender, and seasonality. Analyses aimed to reveal a significant change in expected admission numbers (if any) and/or to examine the admission patterns of NCD patients during the pandemic.

## Materials and Methods

### Data Collection

Data were recruited for patients with NCD diagnoses, with at least one outpatient visit or hospitalization at Hacettepe University Hospitals from January 1, 2018 through June 15, 2021; dependent group analyses were restricted to the period between March 11, 2018 and July 15, 2021 for seasonality control. Patient records were identified using ICD-10 codes matching ischemic heart disease (I-20 to I-25), hypertension (I-10 to I-15), diabetes (E-10 to E-14), cancer (C-00 to C-97), and chronic obstructive pulmonary disease (COPD) (J-44).

**Fig. 1:**
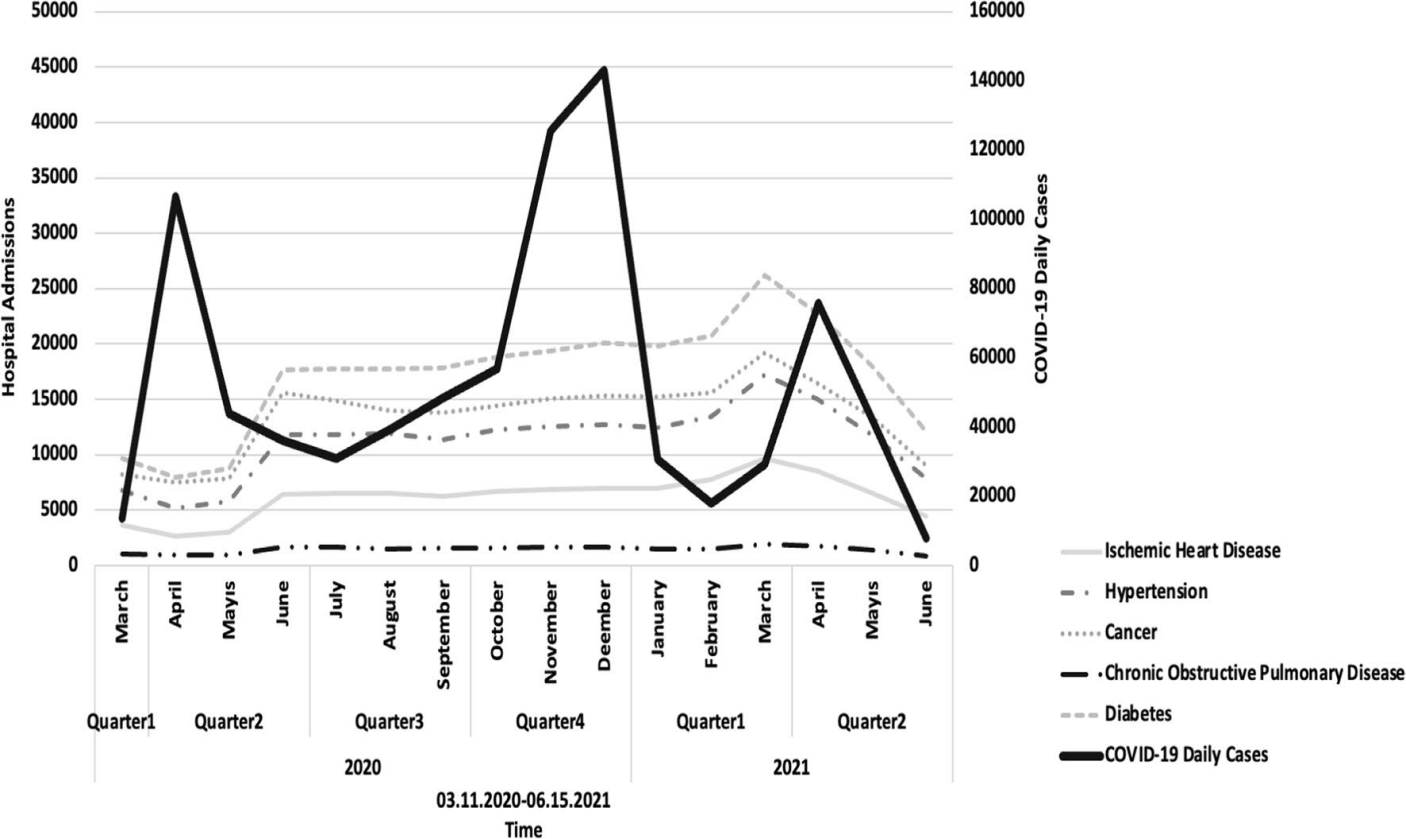
Distribution of admission frequencies to Hacettepe University Hospitals over the pandemic among patients diagnosed with non-communicable diseases and National COVID-19 (New) case numbers in Turkiye over the same timeline

Besides the type of NCDs, data were recruited on age, gender, date and type of admission (ER, in-patient and out-patient), and the clinical departments of admissions. Dates were obtained for recurrent admissions (if any). Daily COVID-19 numbers for Türkiye were obtained from the 'TURCOVID-19' website [6] for comparing distribution of admission frequencies with that of COVID-19 case numbers in the general population (Fig. 1) to investigate whether increase in COVID-19 might have scared patients to visit the hospital.

All hospital admissions (including outpatient visits and hospitalizations) of adult patients (aged 18 or older) with selected ICD-codes were included in analyses for the period from January 1, 2018 to June 15, 2021.

### Ethical Issues

Ethical approval was obtained from Hacettepe University Faculty of Medicine Health Sciences Research Ethics Committee (Approval Code: GO 21/59) and further administrative permissions were obtained from Hacettepe University Hospitals’ management.

### Statistical Analysis

Electronic data were organized and grouped using Microsoft Excel 2020 (Microsoft Corporation. Redmond. USA), using data add-ins and pivot table. Descriptive and comparative analyses together with time series were completed using IBM SPSS v. 28.0 and the open access R and R Studio analysis programs (v. 2021.09.2).

Descriptive statistics included distribution of number and percentages. Normality was tested by visual (histogram and probability graphs) and analytical methods (skewness, kurtosis values, Shapiro–Wilk test and coefficient of variation). Medians and quartiles were presented for variables with non-normal distributions.

Potential variations in admission frequencies, types and patterns of NCD patients were comparatively evaluated for the pandemic period (from March 11, 2020 through June 15, 2021) with the pre-pandemic period (from March 11, 2018 through June 15, 2019); adjustments were made for seasonal variations and potential confounders (age and gender) were. Sensitivity analysis was conducted on a subgroup of admissions (different number of patients each five NCDs), for patients with at least one admission in either of the two study periods, controlling for dependency.

Besides the filtering processes for general applications, additional filters and analyses were used in time series analyses. ARIMA (*p*.*d*.*q*) (Box–Jenkins) models were conducted, where, *p* value indicates the degree of auto regression (AR), *d* = the number of degrees of difference, and *q* = the degree of moving average (MA). The series were made stationary before any operation: the mobility of the series (already stationary or converted to stationary due to seasonality or the presence of a trend by using difference operations) are examined with ACF and partial ACF (PACF) graphs. Models were determined according to the slow decrease of the series and predictions were based on verified models. Analyses were based the rule of at least 50 observations [7]. Time series curves were drawn for each of the five NCDs; seasonality and trend characteristics were determined. ARIMA model was used for predictive values; actual data were inserted into the graph with 95% confidence intervals, and white noise controls were completed. The margin of error for type 1 was set at 0.05 for all analyses.

## Results

Data were recruited for a total of 3,008,871 admissions; of these, 6114 died in the hospital. Number of outpatient admissions were significantly higher than hospitalization frequencies for all NCDs studied, as expected (Table 1).

**Table 1:** Descriptive characteristics of patients with non-communicable diseases admitting to Hacettepe University Hospitals (from January 01, 2018 through June 15, 2021)

Characteristics	Ischemic heart disease	Hypertension	Diabetes	Cancer	COPD
Age (years)					
Mean ± SD	61.6 ± 14.1	59.4 ± 15.2	54.7 ± 16.5	56.6 ± 15.1	62.8 ± 14.8
Median (25%, 75%)	63.0 (54.0, 72.0)	61.0 (50.0, 70.0)	56.0 (44.0, 67.0)	58.0 (47.0, 68.0)	65.0 (54.0–74.0)
Gender, *n* (%)					
Female	16,963 (55.2)	22,989 (42.6)	30,625 (39.7)	23,593 (45.6)	4098 (61.4)
Male	13,768 (44.8)	30,930 (57.4)	46,455 (60.3)	28,144 (54.4)	2577 (38.6)
Total	30 731	53 919	77 080	51 737	6 675
Admission, *n* (%)					
Outpatient	336,693 (83.5)	610,322 (84.0)	838,737 (84.0)	601,783 (77.7)	85,937 (82.0)
Inpatient	66,267 (16.5)	116,336 (16.0)	160,678 (16.0)	173,299 (22.3)	18,819 (18.0)
Total	402,960	726,658	999,415	775,082	104,756
In-hospital death, *n* (%)^a^	775 (2.5)	1268 (2.3)	1573 (2.0)	2 088 (4.0)	410 (6.1)
COVID-19 cases, *n* (%)	520 (1.7)	1074 (2.0)	1912 (2.6)	537 (1.0)	194 (2.9)

*COPD* chronic obstructive lung disease, *SD* standard deviation

^a^Number of patients died during hospitalization

Rates of in-hospital deaths varied between 2.0% (diabetes) and 6.1% (COPD); COVID-19 infection was also more prominent among patients with COPD (2.9%), with the lowest frequencies among cancer patients (1.0%) admitting to Hacettepe Hospital during the pandemic period.

For robust comparisons of admission frequencies during the pandemic period with that of the corresponding pre-pandemic period, the data set was restricted for time span (from March 11, 2018 through June 15, 2019 and from March 11, 2020 through June 15, 2021) and to individuals with at least one admission in both periods. Any patient admitted to the hospital preceding the pandemic was considered to have a preference to choose the same hospital in the pandemic, while those visiting Hacettepe Hospital in the pandemic was excluded from analyses in comparing actual versus “expected” frequencies in time series analysis (Table 2). In the sub-cohort of patients with five selected NCDs, distribution of the actual number of admissions during the pandemic, and over the preceding period, by admission types revealed significantly lower frequencies for all 5 NCDs lower during the pandemic period, compared to actual numbers obtained in the pre-pandemic period (*p* values for Wilcoxon signed-rank test were all < 0.001).

**Table 2:** Comparative admission numbers of NCD patients to Hacettepe University Hospitals during the COVID-19 pandemic and the corresponding time period preceding the pandemic

Admission types and periods^b^	Ischemic heart disease (*n* = 10,475)		Hypertension (*n* = 18,970)		Diabetes (*n* = 27,370)		Cancer (*n* = 17,291)		COPD (*n* = 2156)	
	Median	*p* value^a^	Median	*p* value^a^	Median	*p* value^a^	Median	*p* value^a^	Median	*p* value^a^
Outpatient										
Pre-pandemic	6.0	** < 0.001**	6.0	** < 0.001**	6.0	** < 0.001**	6.0	** < 0.001**	8.0	** < 0.001**
COVID-19 period	3.0		4.0		4.0		3.0		4.0	
Inpatient										
Pre-pandemic	2.0	** < 0.001**	2.0	** < 0.001**	2.0	** < 0.001**	3.0	** < 0.001**	3.0	** < 0.001**
COVID-19 period	1.0		2.0		2.0		2.0		2.0	
Total										
Pre-pandemic	7.0	** < 0.001**	7.0	** < 0.001**	6.0	** < 0.001**	7.0	** < 0.001**	9.0	** < 0.001**
COVID-19 period	4.0		4.0		4.0		4.0		5.0	

COVID-19 period is from March 11, 2020 through June 15, 2021

^a^Comparisons were made for dependent groups, restricting data set to those with at least one admission in both periods. *p* values were obtained from Wilcoxon signed-rank test

^b^Pre-pandemic period is from March 11, 2018 through June 15, 2019

Admission frequencies to various clinical departments by patients with NCDs were compared and contrasted by study periods. When admission frequencies over the COVID-19 pandemic period is compared to the pre-pandemic period by ischemic heart disease patients were examined for different clinical departments, the significant decrease was observed for admissions to internal medicine, geriatrics, family medicine, cardiology and cardiovascular surgery units/departments during the pandemic compared to the numbers in pre-pandemic period (*p* = 0.012 for internal medicine, *p* < 0.001 for others). Patients with hypertension admitted less to internal medicine, geriatrics, family medicine, cardiology and cardiovascular surgery units/departments over the pandemic, compared to frequencies in corresponding pre-pandemic period (*p* < 0.001 for all). Admissions to the emergency department, internal medicine, geriatrics, family medicine, endocrinology, nephrology, ophthalmology, and neurology units/departments decreased significantly in the pandemic period among patients with diabetes (*p* = 0.019 for emergency services, *p* < 0.001 for others). Admissions of cancer patients to internal medicine, geriatrics, family medicine, medical oncology and radiation oncology units/departments significantly decreased during the pandemic (*p* = 0.003 for internal medicine, *p* < 0.001 for others). Frequencies of admissions were significantly lower among COPD patients over the pandemic period to the geriatrics, family medicine, cardiology, and chest diseases units/departments during the pandemic period (*p* < 0.001 for all) (Table 3).

**Table 3:** Statistical significance of change in admission numbers to various clinical departments before and during the COVID-19 period, with regards to selected disease types (Ankara, 2022)

Units and Departments^a^	Ischemic Heart Disease	Hypertension	Diabetes	Cancer	COPD
	*p* value*	*p* value *	*p* value *	*p* value *	*p* value *
Emergency	0.519	0.581	**0.019**	0.741	0.447
Internal medicine	**0.012**	** < 0.001**	** < 0.001**	**0.003**	0.972
Geriatrics	** < 0.001**	** < 0.001**	** < 0.001**	** < 0.001**	** < 0.001**
Family medicine	** < 0.001**	** < 0.001**	** < 0.001**	** < 0.001**	** < 0.001**
Infectious diseases^b^	0.811	0.062	0.526	0.992	0.302
Cardiology	** < 0.001**	** < 0.001**			** < 0.001**
Cardiovascular surgery	** < 0.001**	** < 0.001**			
Endocrinology			** < 0.001**		
Nephrology			** < 0.001**		
Ophthalmology			** < 0.001**		
Neurology			** < 0.001**		
Medical oncology				** < 0.001**	
Radiation oncology				** < 0.001**	
Chest diseases					** < 0.001**

*Only *p* values are presented. Comparisons were made for dependent samples, using Wilcoxon signed-rank test

^a^Since the intensive care data were insufficient for dependent groups in both periods, analysis could not be performed

^b^Applications made only to Infectious Diseases units/departments increased for all diseases during the COVID-19 period. All other significant differences favored a decrease in the pandemic period compared to corresponding pre-pandemic period

Figure 1 presents the admission frequencies to Hacettepe University Hospitals for five NCDs during the first 15 months of the pandemic, with corresponding daily new COVID-19 case numbers in the country over the same timeline. Hospital admission frequencies varied over time with similar trends for all 5 NCDs, irrespective of the daily COVID-19 cases in the nation; trends of NCD admissions were in parallel to restrictions in number of outpatient appointments and hospital beds reserved for non-COVID-19 patients in the hospital over the presented timeline.

**Fig. 2:**
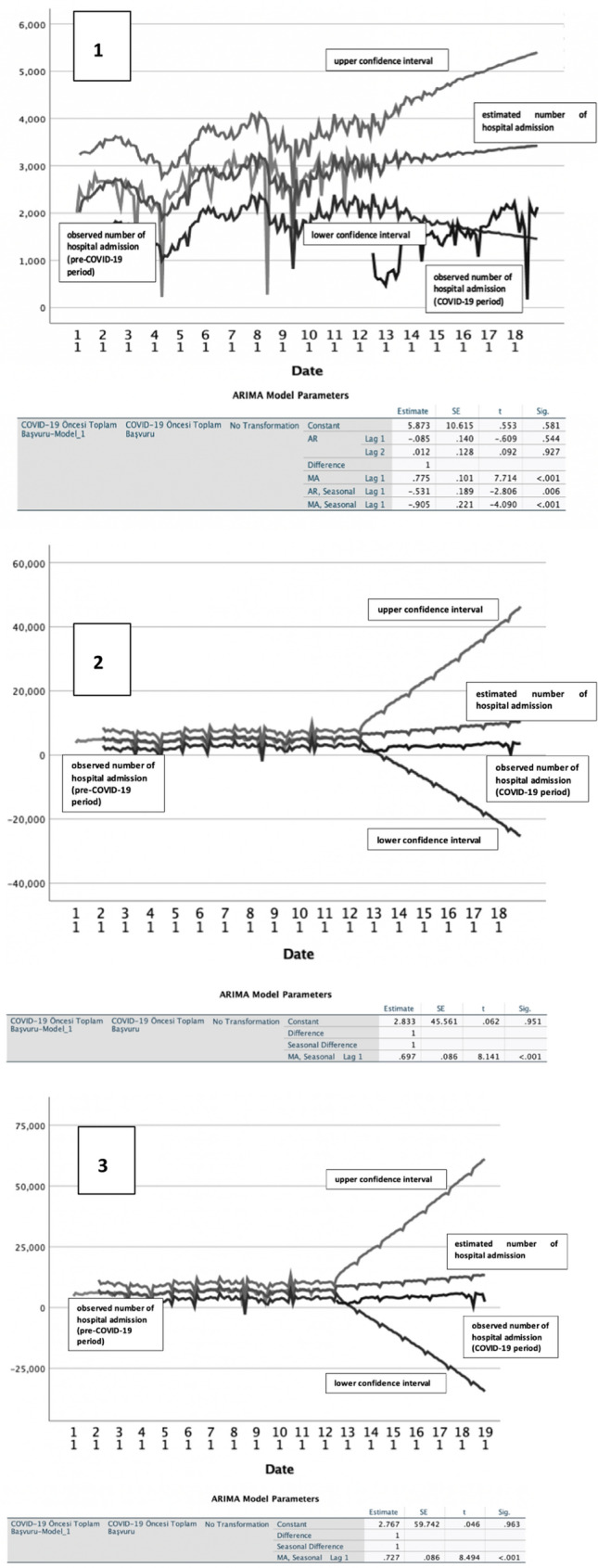

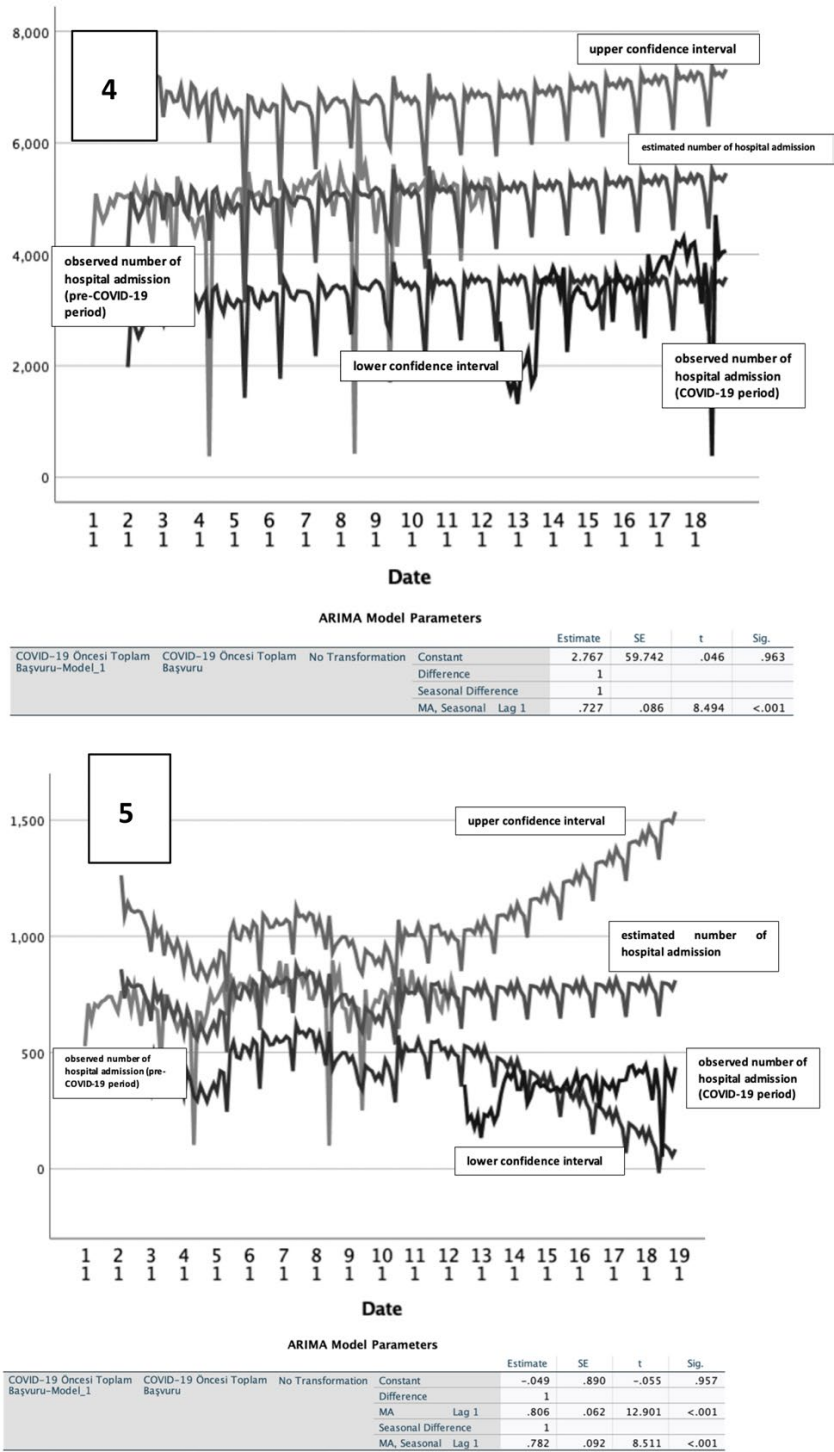
Time series analysis. 1. Ischemic Heart Disease, current ARIMA model; (2,1,1) (1,0,1). 2. Hypertension, current ARIMA model; (0,1,0) (0,1,1). 3. Diabetes, current ARIMA model; (0,1,0) (0,1,1). 4. Cancer, current ARIMA model; (0,0,0) (0,1,1). 5. Chronic Obstructive Pulmonary Disease, current ARIMA model; (0,1,1) (0,1,1)

Time series analyses were performed using weekly admission numbers for the five diseases examined in the pre-pandemic period to forecast expected admission numbers over the corresponding dates over the pandemic period. After seasonal-, and trend-adjustments, admission frequencies over the pandemic period remained below the time series curves estimated for all 5 NCDs studied (Fig. 2). Actual number of admissions for ischemic heart disease cancer and COPD in the pandemic period remained below the lowest 2.5% confidence curves forecasted with the time series analysis.

## Discussion

This study aimed to investigate how adult patients with major NCDs used Hacettepe Hospitals for their routine/required health needs over the pandemic. Secondary data analysis of electronic hospital records might have biased comprehensiveness of the data due to incorrect/incomplete ICD coding, leading to a selection bias, but such an error (if any) is expected to be nondifferential across the pandemic and pre-pandemic periods, with a bias towards the null. Thus, the observed significant decrease in admission frequencies over the pandemic suggests that the “unmet health need” among these patients may lead to increased morbidity, disability, and premature mortality in NCD patients in future years, if they could not get service from other hospitals. The size of individual care demands of NCD patients during the pandemic, and their access to health service providers is beyond the scope of our work. Nonetheless, consistency of our findings with those of the published literature supports a diminished care provision for NCD patients over the pandemic.

In a study examining the number of admissions specific to cardiovascular disease for the years 2017–2020 in Brazil, the decrease in admissions in 2020 were found to be statistically significant (*p* = 0.02) [8]. In a similar study conducted in the USA, hospital admissions for diabetes decreased by 35.8% in April 2020 compared to that in 2019 [9]. A significant decline in hospital admissions was also reported for COPD patients, with a 58% decrease in 2020 compared to 2019 admissions [10]. Rosenbaum reported that immunotherapy, tumor resection and inpatient treatment of cancer patients were adversely affected by the COVID-19 process, for lymphoma patients, in particular. Some treatment modalities were reportedly changed during the pandemic period to decrease hospitalizations; for example, some patients with breast and rectal cancers received systemic therapy before, rather than after, surgery [11].

Long curfews implemented in Turkey over March through May, 2020, and over December through February 2021 might have negatively affected the health care seeking behaviors during the pandemic, among elderly, in particular [12]. A 2-month-long curfew was initiated on March 21, 2020 for individuals aged 65 and over; they were allowed to go out for up to 4 h on Sundays, only, starting from May 10, 2020. Starting from August 13, 2020, curfews were managed by provincial authorities based on local prevention needs, through mid-November 2020 [13], from then on elderly were allowed to go out between 10 AM and 4 PM [14]. With a gradual normalization process over months, all such restrictions were terminated after availability of COVID-19 vaccines in Turkey by mid-January 2021, [15]. Apart from strict, stay-home restrictions, the decrease in the number of hospital admissions over the first year of the pandemic might also be linked with restrictions in public transport use, which appear to be a major mode of transport for patients with low socioeconomic status, and the elderly. Average age of the study population varied between 54 and 63 years, suggesting about half of the study population was affected from "stay at home" and public transportation bans. Together with individuals' reluctance to go out due to fear of COVID-19 and/or restricted number of appointments spared in hospitals for regular care visits during the pandemic [16], public bans might have avoided patients with NCDs from applying to health services.

Hacettepe University Hospitals are well-known tertiary care centers, and a significant number of patients with chronic conditions come from a variety of different cities. Such patients could have used local hospitals during the pandemic to avoid long drives/trips, especially for routine, simple visits/procedures. That is, decreased admission numbers do not preclude the possibility of getting timely and effective care from another care setting. Unfortunately, revealing such a deviance in case-profile was beyond the scope of this study and was unethical given national regulations on patient electronic data safety and security prevents use of national health records.

It is noteworthy that many hospitals in the country have become pandemic hospitals with the increase in the number of COVID-19 cases. Elective procedures were delayed during this period, and many existing patient appointments were either cancelled/postponed. The only exception was for cancer patients; both in and outpatient services provided for cancer were kept almost stable over the pandemic [17], with some modifications in treatment schemes, by postponement of elective surgeries and/or replacement of unnecessary surgeries with conservative medical treatments. Within the scope of our observations and verbal interviews with patients and care givers, the management of oncology hospital of Hacettepe University tried special care modifications not to neglect and victimize any patient for any reason; having a separate building eased this process. Thus, we assume that the relative decrease in cancer patients’ admissions in the pandemic could be mainly be due to preference of patients to have their regular visits/chemotherapy sessions in local hospitals, if possible.

The Ministry of Health can anonymously investigate, by working in partnership with academicians and scientist, the burden of unmet need (if any) for management of NCDs over the pandemic. Such an investigation would be invaluable to forecast the need in future years for combating belated diagnosis of NCDs, burden of complications due to delayed/insufficient care services to NCD patients, and/or to distinguish these possibilities from additional burden of long-COVID-19 on chronic health problems.

When department-based admissions made by patients with ischemic heart disease, hypertension, and diabetes to the hospital were examined in detail, the profile suggested that expected, regular check-ups were disrupted in the pandemic, making patients prone to advanced/worsened disease states. Lack of difference in emergency service admissions suggests that COPD patients preferred to use emergency department for acute or seasonal exacerbations, while skipping routine checkups. Absence of a statistically significant decrease in emergency department admissions in this study for all four NCDs, except for diabetes, is in contrast to published work on similar topics [18–20], which, at least partially, can be explained by non-restrictive use of emergency room over the pandemic.

Time series models were used to investigate disease-specific trends, and seasonality patterns (if any). Similar models are used in literature for non-communicable diseases and found to have good predictive ability if the model is set up correctly [21]. In our cohort, expected patient admissions in pandemic period was predicted based on pre-pandemic period admissions using appropriate ARIMA models, and their graphs were drawn for visual comparisons over 15 months of the pandemic. Time series models with 95% confidence intervals revealed a significant decrease in all NCD-related admissions over the pandemic to levels below 2.5% of the expected admissions line. Although the temporal length of this decline differed for diseases, all admissions decreased considerably over the pandemic. The COVID-19 process curve for ischemic heart disease was longer and deeper below the lower confidence limit than for other diseases. A similar time series curve was observed for COPD, but the decrease in admissions were relatively less significant. Process curves were shorter for hypertension and diabetes, whilst cancer had the most stable curve in time series models. Shorty, with subtle differences across diseases, pandemic period negatively affected patient admissions for all five diseases, suggesting delays/disruptions in case management, follow-up, treatment and/or rehabilitation processes over the first 15 months of the pandemic in Turkey.

Study has certain limitations. Research in a single hospital and for five diseases limit generalizability of our findings. Analysis on the stage or severity of the disease on admission was beyond the scope of this study, given relevant data were not accessible. The significant change in percentages of hospitalization, intensive care stay and in-hospital deaths over the pandemic could be used as a surrogate measure for disease severity but these data were not complete, either. We calculated in-hospital deaths for patients admitting to Hacettepe Hospitals with diagnoses of any of the five NCDs over the study period, yet, a robust evaluation of the impact of the pandemic on disease-specific death rates was not possible. Also, the potential size of survival bias hindering admissions, the role of competitive deaths and deaths after hospital discharge could not be estimated with available data. Thus, the obtained decreases in admission rates can be considered as the “minimum” burden of the pandemic on proper health service provision for selected noncommunicable diseases. Lastly, the sets of ICD-10 codes used to identify hypertension, diabetes, COPD, ischemic heart disease and cancer admissions were quite large, yet, we might have missed some cases due to incorrect coding, not to mention the potential for selection bias due to improper coding at the time of admission. It is (if any), however, expected to be nondifferential across the time periods.

## Conclusion

Literature reveals that pandemic conditions have been associated with disruptions in both hospitalizations and outpatient health care services for many diseases, due to overwhelming care needs for COVID-19 cases, with elderly being in the highest risk group for adverse health outcomes. Patients with NCDs faced significant problems in getting sufficient medical support for their regular follow-ups, treatment and rehabilitation services, whilst several new NCD cases were likely remained undiagnosed, or diagnosed late. Patients with NCDs had more severe disease courses if infected with COVID-19, compared to other individuals. Accordingly, the anxiety and fear of NCD patients to seek help from any health institution during the pandemic might have avoided admissions for uncrucial needs. Long curfews, public transport restrictions, closures in intercity transits might have prevented several patients from reaching health care in a timely manner. Hospitals restricted daily appointment numbers over the pandemic and the number of hospital beds were mainly used COVID-19 patients, avoiding proper care and health services for many.

The unmet care need over the pandemic for noncommunicable diseases may lead to increase in severity and complications of non-communicable diseases in long run. This study has a pioneer role in investigating the negative effects of the pandemic on timely and comprehensive management of NCDs. Our findings reveal a significantly decreased admissions to both in-patient and out-patient services for diabetes, hypertension, ischemic heart disease, hypertension, and cancer. Given the size of these five diseases in national disability-adjusted life years (DALYs), adverse effects on patient care over the pandemic may lead to significant DALY losses in the next few years. Similar studies in other settings, with further information on outcome of patients after discharge, and use of other resources for health care (if any); data on personal interviews for reasons underlying decrease in admissions (such as, problems with access, fear of infection, etc.) are clearly warranted for conclusive evidence. Robust estimates of the pandemic burden on NCDs require additional information on premature deaths, interaction of COVID-19 with comorbidities, and behavioral factors on prognosis of patients with NCD.

Prospective patient cohorts are clearly warranted for complete and accurate queries for opportunity costs of COVID-19 care on NCD management over the pandemic and additional qualitative research will be valuable to reveal patients’ perspective on their care needs, and alternative care modalities (if any).

## Data Availability

Data used and analyzed in this study will be promptly available for the publisher upon request.

## Abbreviations

NCD: Non-communicable diseases
ICD-10: International Classification of Diseases 10th Revision
COPD: Chronic obstructive pulmonary disease
ER: Emergency room
USA: United States of America
IBM: The International Business Machines
SPSS: Statistical Package for the Social Sciences
AR: Auto regression
MA: Moving average
COVID-19: Coronavirus disease 2019
SD: Standard deviation
